# Educational Attainment and HIV Prevalence by Age Among Pregnant Women in South Africa

**DOI:** 10.1007/s10461-026-05035-3

**Published:** 2026-01-27

**Authors:** Chibuzor M. Babalola, Kalpana Gopalkrishnan, Mandisa M. Mdingi, Freedom Mukomana, Msindisi Gqirana, Christina A. Muzny, Christopher M. Taylor, Remco P. H. Peters, Andrew Medina-Marino, Jeffrey D. Klausner

**Affiliations:** 1 Department of Population and Public Health Sciences, Keck School of Medicine University of Southern California, 1845 N Soto, Los Angeles, CA 90032 USA; 2 Research Unit, Foundation for Professional Development, East London, South Africa; 3 Division of Infectious Diseases, University of Alabama at Birmingham, Birmingham, USA; 4 Louisiana State University Health Sciences – New Orleans, Louisiana, USA; 5 Perelman School of Medicine, University of Pennsylvania, Philadelphia, USA

**Keywords:** HIV prevalence, HIV prevention, Adolescent girls and young women, Pregnancy, socio-behavioral factors, Educational attainment

## Abstract

We examined the association between educational attainment and HIV positivity among pregnant women in a high HIV-prevalence setting and assessed how this relationship varies by age to inform targeted prevention strategies. This cross-sectional study included 2003 pregnant women aged 21–44 years attending their first antenatal visit (<27 weeks’ gestation) at four public health facilities in East London, South Africa, between March 2021 and May 2024. Educational attainment was categorized as pre–high school (< grade 10), high school (grades 10–12), diploma (post–high school), or degree (associate’s or bachelor’s). Age was categorized into four groups (21–24, 25–29, 30–34, and 35–44 years). HIV status was determined through routine antenatal testing. We used logistic regression to assess associations between educational attainment and HIV positivity, adjusting for age, partner’s HIV status, and participant sexually transmitted infection (STI) status. Overall HIV prevalence was 31.0% (95% CI, 28.9%–33.0%). Compared with women with less than a high school education, the odds of HIV infection were lower among women who attained high school education (adjusted odds ratio [AOR], 0.59; 95% CI, 0.40–0.87), a diploma (AOR, 0.40; 95% CI, 0.24–0.67), or a degree (AOR, 0.21; 95% CI, 0.09–0.43). However, this inverse association was not observed among women aged 35–44 years. In conclusion, higher educational attainment was associated with lower HIV prevalence among pregnant women, but this protective association diminished with increasing age. HIV prevention strategies should account for both socioeconomic factors and age-related interpersonal dynamics influencing HIV vulnerability.

## Introduction

In sub-Saharan Africa, cisgender women and girls of reproductive age account for a staggering six out of 10 new HIV infections [1]. In South Africa, HIV prevalence among pregnant women remains alarmingly high, often exceeding that of the general adult population [2]. In 2022, antenatal HIV prevalence varied from 15% to 33% across the country’s nine provinces [3]. Research indicates that HIV acquisition more than doubles during pregnancy and the postpartum period [4]. The increased healthcare utilization during pregnancy could be leveraged to enhance HIV prevention efforts and monitor health outcomes among women of reproductive age in high-prevalence settings [5]. 

Despite the proven effectiveness of daily oral pre-exposure prophylaxis (PrEP) in reducing HIV transmission, coverage among pregnant women in South Africa is low at just 6.5% [3]. Beyond structural factors, societal influences, and knowledge gaps, adherence issues could significantly challenge the effectiveness of oral PrEP in the broader population [6, 7]. Therefore, comprehensive HIV prevention strategies extending beyond biomedical interventions remain vital. Socioeconomic factors, such as poverty and limited education, often exacerbate systemic inequities, heightening sexual and reproductive health risks for young women [8, 9]. These challenges can manifest in inequitable partnerships and power imbalances, complicating the design of effective, tailored interventions [10, 11]. 

Post-secondary education may provide a protective effect against HIV risk for women in sub-Saharan Africa, but this relationship is underexplored, and the indicators to guide intervention design or impact measurements are not well established [12]. Initiatives focused solely on subsidizing formal education or lengthening stay in secondary school may fall short in addressing deeper issues like financial instability and contextual employment limitations [11, 13]. Conditional cash transfers linked to education may alleviate financial barriers while supporting educational attainment [14], however other entrenched inequities or relational dynamics that drive social vulnerability and reduce sexual and reproductive health agency may still prevail in certain cohorts [10]. 

This study aimed to enhance the understanding of how socio-behavioural HIV prevention strategies could be more effectively implemented across the reproductive lifespan of women in South Africa. We examined the relationship between educational attainment and HIV infection among pregnant women in a South African community, and its variation by age to assess how population dynamics can inform targeted interventions that address the unique vulnerabilities of different age cohorts.

## Methods

### Study Design and Setting

We conducted a cross-sectional analysis using data from the Philani Ndiphile study, a clinical trial in East London, Buffalo City Metropolitan Municipality, Eastern Cape province, South Africa. The Philani Ndiphile study, NCT04446611, is a three-arm (1:1:1) hybrid effectiveness-implementation trial evaluating the impact of diagnostic screening algorithms for curable sexually transmitted infections (STIs) on adverse birth outcomes. In this report, we refer to the parent study as “Philani.”

Buffalo City is the second-largest municipality in the Eastern Cape, the fourth most populous province in South Africa. According to the 2022 national antenatal survey, HIV prevalence among pregnant women in the Eastern Cape was 32.9% (95% CI, 31.5%-34.2%) [3], the second highest among South Africa’s nine provinces. According to Statistics South Africa (Stats SA) data, the Eastern Cape province faces the most significant socioeconomic disparities and is characterized by the highest unemployment rates, and lowest levels of educational attainment [15]. 

In the Philani parent study [16], pregnant women aged 18 years or older and attending their first antenatal visit before 27 weeks’ gestation, were enrolled from four public health facilities starting in March 2021. Participants were randomized to one of three arms: (1) STI screening with point-of-care nucleic acid amplification tests (NAATs) at enrolment, followed by tests of cure for individuals with positive test results; (2) STI screening with NAATs at enrolment and rescreening in the third trimester; or (3) syndromic management, the current standard of care in the absence of diagnostic resources, where empiric antibiotics are provided to pregnant women who report urogenital symptoms [17]. The STIs screened for included *Chlamydia trachomatis*, *Neisseria gonorrhoeae*, and *Trichomonas vaginalis*.

### Participants

This cross-sectional analysis used enrolment data from Philani, collected at the first antenatal visit (before 27 weeks’ gestation). The source data included participants enrolled between March 2021 and May 2024, irrespective of trial intervention. At enrolment, all participants received standard antenatal care per South African guidelines, including universal syphilis screening and HIV testing [17]. Syphilis screening was conducted via a rapid diagnostic treponemal test (syphilis RDT), followed by a rapid plasma reagin (RPR) test for those who tested positive. Women diagnosed with HIV at the first antenatal visit, who were not already on antiretroviral therapy (ART), were initiated on treatment. Nurse midwives performed pelvic examinations and obtained obstetric histories at this visit.

### Data Collection

At the first antenatal visit, trained research nurses administered an enrolment questionnaire, capturing data on socioeconomic status (including income and education), demographics, sexual and behavioural history, medical history, and past obstetric history. Data were securely stored in a Health Insurance Portability and Accountability Act (HIPAA)-compliant REDCap database, hosted by the Foundation for Professional Development in East London, South Africa.

### Ethics

The Philani study was approved by the University of Cape Town, Faculty of Health Sciences Human Research Ethics Committee (reference 676/2019) and operated under a reliance agreement with the University of Southern California Institutional Review Board (HS-21-00244). Written informed consent was obtained from all participants.

### Variables and Measurements

The primary outcome was *HIV infection status*, categorized as living with HIV or HIV-uninfected, based on HIV test results.

The primary exposure variable was *educational attainment*, defined by the highest level of formal education completed. In the source data, it was originally categorized into five levels: (I) less than grade 10 of secondary school (equivalent to less than senior high school), (II) grades 10 or 11 of high school, (III) grade 12 of high school, (IV) a national diploma (a post-secondary qualification below a bachelor’s degree), and (V) a degree (associate’s or bachelor’s equivalent). For analysis, these were recoded into four categories: (I) pre-high school (< grade 10), (II) high school (grades 10-12), (III) diploma, and (IV) degree.

The primary covariate was *age*, analysed continuously and in four groupings (21-24, 25-29, 30-34, 35-44 years), chosen to reflect distinct life stages with potential variations in sexual behaviour, relationship stability, and health information access. Pregnant women younger than 21 years of age were excluded to focus on participants likely to have completed minimum formal education.

### Statistical Analysis

We primarily examined the age-adjusted relationship between educational attainment and living with HIV at the first antenatal visit. Descriptive analyses summarized participant characteristics. Pearson’s Chi-squared tests statistically assessed differences across levels of educational attainment.

HIV prevalence was calculated as the proportion of pregnant women living with HIV, both within the overall sample and within exposure categories.

We used multivariable logistic regression to estimate the association between educational attainment and HIV status. Model 1 A adjusted for age as a continuous variable, while Model 1B included additional covariates. In the fully adjusted model, we incorporated three covariates selected based on evidence from the scientific literature and descriptive analysis. We prioritized relevance to HIV risk and educational attainment, while ensuring no issues with multicollinearity. Specifically, we included the participant’s report of their partner’s HIV status, the participant’s clinically confirmed current STI status (defined as a diagnosis of syphilis, *Chlamydia trachomatis*, *Neisseria gonorrhoeae*, or *Trichomonas vaginalis*), and whether the participant personally earned some monthly income versus none, as an indicator of financial agency [8, 18–20]. Finally, we conducted stratified logistic regression (Model 2) to estimate age group-specific associations of educational attainment with HIV. This analysis was left unadjusted to avoid overfitting across age and education strata, and age stratification was also expected to capture much of the variability across groups.

Statistical analyses were conducted using R, version 4.4.1 (R Foundation for Statistical Computing, Vienna, Austria), 2024. Statistical significance was 2-tailed at α = 0.05. Odds ratios with 95% Confidence Intervals (CIs) were ascertained in the logistic regressions.

## Results

The source dataset included 2,249 pregnant women aged 18-44 years. After excluding women younger than 21 years (*n*=244) and those with missing data on educational attainment (*n*=2), the final analytic sample consisted of 2,003 women aged 21-44 years.

### Demographics

The median gestational age at the first antenatal visit was 14 weeks (interquartile range [IQR]: 4-24 weeks), and the median age of the cohort was 29 years (IQR: 21-38 years). Most participants were in or had completed high school (78.8%, *n*=1578), with fewer holding a higher education as diploma (9.9%, *n*=199) or degree (4.3%, *n*= 87); or attaining less than a high school education (6.9%, *n*=139).

Highlighting the notable patterns in Table 1, age distribution varied significantly by educational attainment, with the oldest age group (ages 35-44) being prevalent among those with less education (28.8% at pre-high school level), while relatively more younger women (ages 25-29) had a diploma (37.2%) or degree (34.5%). Nearly half (51%) of the cohort reported no personal monthly income, significantly more among those with less than a high school education (66.2%). In contrast, while only very few overall (2.5%) were at the highest monthly income level of >10,000 ZAR/550 USD/530 Euros), degree holders were most represented in this group. Personal income was more commonly the primary source of household income among diploma (54.3%) or degree (49%) holders, whereas partner and other income sources were more common for those with a less than a high school education (71.2%).

Showing negligible variation by educational attainment, most participants (93.5%) reported being in a steady relationship or married, having only one recent sexual partner (88.6%), and “no condom use” at their last sexual encounter (90.3%). For elements suggestive of relational vulnerability, few participants (9.7%) reported engaging in sex against their will, and though this not statistically significant, the higher proportions were among those with higher educational attainment (18% diploma or 16.1%-degree holders) compared to those with lower educational attainment (9.3% high school or 9.7% pre-high school). In contrast, all participants who reported engaging in sex for financial benefit (1%) had lower educational attainment.

By participant’s STI status and knowledge of or partner’s HIV status, STI positivity at the first antenatal visit (18%) was significantly higher among pregnant women with less than a high school education compared to those with a degree (26.6% vs. 10.3%). Degree holders were also the most likely to know their partner’s HIV status and report the lowest proportion of partners living with HIV. No degree holder reported having a partner with HIV who was not on antiretroviral therapy (ART).

**Table 1:** Participant characteristics by educational attainment, at first antenatal visit, March 2021 to May 2024; *N* = 2003 pregnant women aged 21 to 44 years in the philani Study, East London, South Africa

	Total (*N*=2003)		Pre-High School (*n*=139)		High School (*n*=1578)		Diploma (*n*=199)		Degree (*n*=87)		
	*n*	%	*n*	%	*n*	%	*n*	%	*n*	%	*p*-Value
Age, years											0.029
21 to 24	428	21.4	26	18.7	349	22.1	41	20.6	12	13.8	[χ^2^(9) = 18.54]
25 to 29	615	30.7	36	25.9	475	30.1	74	37.2	30	34.5	
30 to 34	563	28.1	37	26.6	440	27.9	55	27.6	31	35.6	
35 to 44	397	19.8	40	28.8	314	19.9	29	14.6	14	16.1	
Monthly personal income											<0.001
No current personal income	1070	53.4	95	68.3	859	54.4	76	38.2	40	46.0	[χ^2^(9) = 210.29]
Less than 5000 ZAR / 275 USD	708	35.3	39	28.1	582	36.9	72	36.2	15	17.2	
5001 to 10,000 ZAR / 550 USD	174	8.7	4	2.9	121	7.7	35	17.6	14	16.1	
More than 10,000 ZAR / 550 USD	51	2.5	1	0.7	16	1.0	16	8.0	18	20.7	
Primary source of household income											<0.001
Personal income	826	41.2	40	28.8	635	40.2	108	54.3	43	49.4	[χ^2^(6) = 28.53]
Partner income	503	25.1	49	35.2	400	25.3	36	18.1	18	20.7	
Other sources (e.g., grants)	674	33.7	50	36.0	543	34.4	55	27.6	26	29.9	
Relationship with current partner(s)											0.386
No current partner	52	2.6	1	0.7	41	2.6	7	3.5	3	3.5	[χ^2^(9) = 9.58]
Casual relationship(s)	79	3.9	4	2.9	70	4.4	3	1.5	2	2.3	
Married or steady relationship with partner	1872	93.0	134	96.4	1467	92.9	189	95.0	82	94.3	
Number of sexual partners, past 6 months											0.816
One	1174	88.6	123	88.5	1393	88.3	179	89.9	79	90.8	[χ^2^(3) = 0.94]
More than one	229	11.4	16	11.5	185	11.7	20	10.1	8	9.2	
Suspects partner may have other partners											0.254
No	1051	52.5	75	54.0	810	51.3	117	58.8	49	56.3	[χ^2^(6) = 7.79]
Yes	547	27.3	39	28.1	448	28.4	42	21.1	18	20.7	
Uncertain or Unknown	405	20.2	25	18.0	320	20.3	40	20.1	20	23.0	
Used condom at last sex											0.873
No	1808	90.3	130	93.5	1418	89.9	180	90.5	80	92.0	[χ^2^(6) = 2.46]
Yes	194	9.7	9	6.5	159	10.1	19	9.5	7	8.0	
Recently agreed to sex against will ^†^											0.560
No	1808	90.3	124	89.2	1430	90.6	181	91.0	73	83.9	[χ^2^(6) = 4.88]
Yes	194	9.7	15	10.8	147	9.3	18	9.0	14	16.1	
Recently agreed to sex for financial benefit											0.181
No	1983	99.0	136	97.8	1561	98.9	199	100.0	87	100.0	[χ^2^(3) = 4.88]
Yes	20	1.0	3	2.2	17	1.1	0	0.0	0	0.0	
Tested positive for an STI at antenatal visit^‡^											0.002
No	1642	82.0	102	73.4	1288	81.6	174	87.4	78	89.7	[χ^2^(3) = 14.57]
Yes	361	18.0	37	26.6	290	18.4	25	12.6	9	10	
Partner is living with HIV											<0.001
Unknown	924	46.1	86	61.9	725	45.9	85	42.7	28	32.2	[χ^2^(9) = 39.18]
No	881	44.0	36	25.9	690	43.7	99	49.7	56	64.4	
Yes, and on antiretroviral therapy	174	8.7	14	10.1	145	9.2	12	6.0	3	3.4	
Yes, and NOT on antiretroviral therapy	24	1.2	3	2.2	18	1.1	3	1.5	0	0.0	

HIV, Human Immunodeficiency Virus; STI, Sexually Transmitted Infection

^†^Response missing from one participant

^‡^Based on rapid treponemal testing for syphilis and/or Gene Xpert test for *C. trachomatis*, *N. gonorrhoeae*, and *T. vaginalis*

χ^2^ = Pearson’s chi-square test statistic; values in parentheses indicate degrees of freedom

### HIV Prevalence

**Fig. 1:**
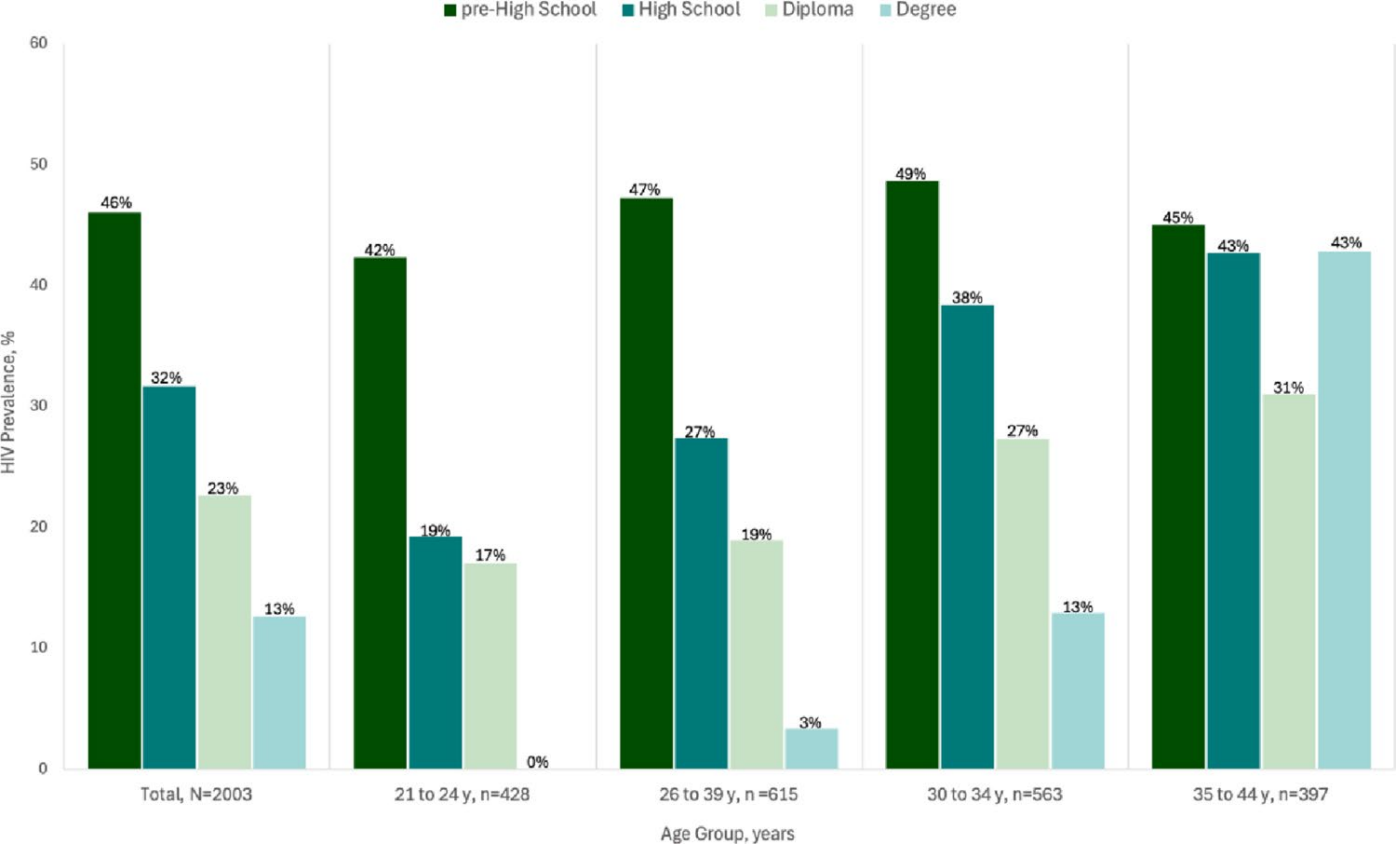
Distribution of participants living with HIV by educational attainment, overall and by age group

The HIV prevalence in the overall sample of pregnant women aged 21-44 years was 31.0% (95% CI, 28.9–33.0), with 620 women testing positive at the first antenatal visit. Illustrated in Figure 1, HIV prevalence was highest among those with the lowest educational attainment (46%), and lowest among those with the highest educational attainment (13%). This pattern held across most age groups, except the oldest aged 35-44 years, where prevalence among degree holders (43%) matched that of those with less than a high school education.

### Relationship Between Educational Attainment and HIV, by Age

Table 2 shows the adjusted odds ratios (AORs) from logistic regressions, consistent across models adjusted for age only (Model A) and additional covariates (Model B).

Compared to pregnant women with less than a high school education, the odds of living with HIV were 41% lower for those with a high school diploma (AOR = 0.59; 95% CI, 0.40-0.87), 60% lower for those with a diploma (AOR = 0.40; 95% CI, 0.24-0.67), and 79% lower for degree holders (AOR = 0.21; 95% CI, 0.09-0.43).

Older age was associated with higher odds of living with HIV, increasing by 7% for each additional year (AOR = 1.07; 95% CI, 1.05-1.09). For additional covariates (Model B), women whose partners were living with HIV had 16 times higher odds of having HIV themselves (AOR = 16.10; 95% CI, 10.8-24.8), while women testing positive for an STI had twice the odds (AOR = 2.07; 95% CI, 1.59-2.68).

**Table 2:** Age-adjusted associations between educational attainment and living with HIV as of the first antenatal visit

	Model A				Model B		
	AOR	95% CI	*P*		AOR	95% CI	*P*
Educational Attainment							
Pre-High School	Reference Education Category				Reference Education Category		
High School	0.58	040-0.83	0.003		0.59	0.41-0.87	0.008
Diploma	0.37	0.23-0.60	<0.001		0.41	0.24-0.70	<0.001
Degree	0.17	0.08-0.35	<0.001		0.21	0.09-0.44	<0.001
Age (Continuous, 21 to 44 years)	1.08	1.06-1.09	<0.001		1.07	1.05-1.09	<0.001
Participant’s STI Status (Tested positive vs. Not)^†^	–	–	–		2.07	1.59-2.68	<0.001
Partner HIV Status (Living with HIV vs. Not or Unknown)	–	–	–		16.0	10.8-24.5	<0.001
Personal Monthly Income (No Income vs. Some Income)	–	–	–		0.96	0.77-1.19	0.7

AOR, Adjusted Odds Ratio; HIV, Human Immunodeficiency Virus; STI, Sexually Transmitted Infection

^†^STIs: Syphilis, C. trachomatis, N. gonorrhoeae, or T. vaginalis

**Fig. 2:**
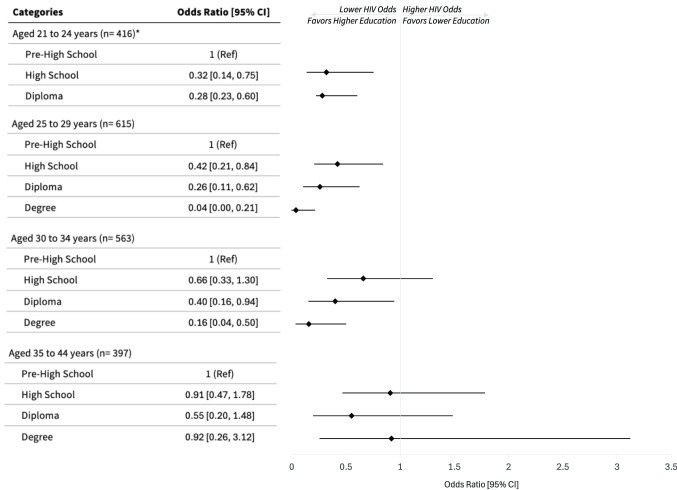
Age group stratified associations between educational attainment and living with HIV as of the first antenatal visit. *The degree category was excluded from the 21 to 24 years age group due to zero participants living with HIV in this category, to avoid unreliable estimates in the model’s fitted probabilities

Figure 2 presents the unadjusted age group stratified associations between education levels and HIV status, to breakdown the dose-response interaction patterns of HIV and education.

In the youngest age group (21–24 years), compared to pregnant women with less than high school education, those with a high school education had lower odds of living with HIV (OR = 0.32, 95% CI: 0.14–0.75), with diploma holders exhibiting even lower odds (OR = 0.28, 95% CI: 0.23–0.60). Among women aged 25–29 years, odds of HIV positivity were progressively lower with increasing educational attainment: high school (OR = 0.42, 95% CI: 0.21–0.84), diploma (OR = 0.26, 95% CI: 0.11–0.62), and degree holders showing the strongest reduction (OR = 0.04, 95% CI: 0.00–0.21). In the 30–34-year age group, the association of educational attainment and HIV status was less pronounced, with wider confidence intervals. Women with high school education showed reduced odds compared to those with less than high school education, though not statistically significant (OR = 0.66, 95% CI: 0.33–1.30). However, progressively lower odds were observed among diploma holders (OR = 0.40, 95% CI: 0.16–0.94) and degree holders (OR = 0.16, 95% CI: 0.04–0.50). In the oldest group (35–44 years), higher educational levels—high school, diploma, or degree—did not demonstrate a clear association with HIV status compared to the pre-high school level.

## Discussion

This cross-sectional analysis of 2,003 pregnant women aged 21 to 44 years in East London, South Africa, revealed a high HIV prevalence (31%) and showed that higher educational attainment was associated with lower odds of living with HIV. The association was strongest among younger women (aged 21-30 years), where having a diploma or degree was consistently linked to reduced odds of HIV infection. However, this relationship weakened for women over 30, becoming less evident in the oldest age group (35-44 years).

Previous studies have offered mixed findings regarding the relationship between education and HIV risk [21, 22]. Early research, especially before the early 2000s, suggested no association or even a reverse effect [23–25], where education was linked to increased risk, possibly due to delayed marriage or increased premarital sexual activity. More recent evidence, especially from sub-Saharan Africa, align with our findings, suggesting that education has a protective effect on HIV risk [26–28]. This trend could be attributed to increased access to HIV prevention information, improved socioeconomic status as observed in our cohort [9, 29], and greater autonomy in sexual and reproductive health decisions among more educated women.

The role of age in modifying the positive impact of education on HIV is less well studied. In our cohort, the favourable influence of higher education became negligible among the oldest age group (35-44 years), where HIV prevalence among degree holders was like that of women with less than a high school education. One study from India [30] broadly found a similar trend, analysing two main age groups (15-24 and 25-50 years) and showing that the reduced likelihood of HIV with educational exposure declined after age 25. Notably, this trend may reflect the cumulative nature of HIV exposure, as individuals who acquire HIV at younger ages remain positive into older age groups. However, variations in healthcare access, established behavioural patterns, and age-specific socioeconomic or relational factors could further limit the benefit of education on HIV risk in older women [31]. Shifting societal dynamics and advancements in HIV awareness and prevention may also explain the observed patterns. Such as if older women faced different risk landscapes earlier in life and were less likely to be tested, while younger women with higher education may have benefited from more recent public health efforts. These historical and contextual factors suggest that further research into age- and cohort-specific influences is necessary. Tailored prevention efforts that address these age-specific challenges are critical to maintaining the benefits of education across the reproductive lifespan [31]. 

Inferring from the socioeconomic dynamics of our study cohort, it was as expected that educational attainment will be significantly correlated with income. However, it is worth noting that most participants (97%) earned less than $550 per month, and more than half depended on partner income or grants to supplement household expenses. This persistent low-income effect may have contributed to dampening the impact of education as age increased. Moreover, we appreciate that the age distribution by educational attainment was not entirely linear; some younger women with higher education contrasted with older women who commonly had lower education levels. This complex age-related socioeconomic pattern could reflect broader trends in which women with higher education in this setting tend to have pregnancies at a younger age, or a few demographic biases during study enrolment in this predominantly low-income population. Older women with higher education might be less incentivized to participate due to greater financial stability, whereas younger women, even those with diplomas or degrees, might still seek study compensation. Additionally, historical factors like fewer educational opportunities for older women could contribute to the observed differences. These contextual trends highlight the importance of designing applied studies that account for such variations.

Our findings also suggested complexities in the relationship between education and sexual and reproductive health agency. While higher education was associated with greater awareness of partner HIV status and fewer partners with HIV, a paradox emerged: some highly educated women reported recently (within 6 months) experiencing elements of sexual coercion, expressed as agreeing to sexual intercourse against their will, though none reported engaging in transactional sex. This picture suggests that while women with higher education may benefit from improved health awareness, they can still face non-economic vulnerabilities, particularly in relational power dynamics or emotional coercion. In contrast, women with lower education levels may be more influenced by financial pressures in their sexual decision-making.

Notably, Women with higher education could be uniquely susceptible to emotional control by partners who perceive a power imbalance, especially if they are financially stable [32]. A study in Uganda [33] found that while higher income generally protected against intimate partner violence in their cohort of married or cohabiting women, those who earned more than their partners faced an increased risk of such violence [33]. Thus, while higher educational attainment could enhance women’s health awareness and socioeconomic status, it may not fully shield them from all forms of sexual risk.

Understanding these dynamics can inform the timing and layering of interventions, such as educational, socio-behavioural, and economic programs, to enhance HIV prevention and control. DREAMS (Determined, Resilient, Empowered, AIDS-free, Mentored, and Safe) is an initiative launched in 2014 through a partnership between the United States President’s Emergency Plan for AIDS Relief, private sector organizations, and other key stakeholders [34]. It represents a comprehensive package of interventions designed to address the structural, behavioral, and biomedical drivers of HIV vulnerability among adolescent girls and young women in sub-Saharan Africa. The program’s education-focused components range from school-based HIV prevention curricula to community-level efforts that promote health literacy and financial empowerment [34]. While DREAMS has demonstrated potential in reducing HIV risk, particularly through increased HIV knowledge and social support, its early impacts in South Africa have been less pronounced compared to other countries like Kenya [35]. These differences are likely due to variations in implementation, community context, and the baseline HIV awareness of the target populations [35]. There are also challenges with sustaining gains across different age groups and socioeconomic settings. However, our findings support the value of such layered interventions, suggesting that DREAMS-like programs could strengthen impact by tailoring strategies to the nuanced vulnerabilities by age within context. Integrating qualitative approaches to address relational power dynamics, alongside economic empowerment and age-specific life-course challenges, could help sustain the positive effect of education, especially for older cohorts.

### Limitations

Our analysis assessed HIV prevalence rather than incidence, necessitating caution in interpreting HIV risk, particularly in older age groups. The cumulative nature of HIV as a lifelong condition means that prevalence estimates in older age groups may reflect earlier infections rather than current transmission dynamics. Nonetheless, the consistent trend of educational protection on HIV among younger women highlights its critical role in reducing HIV risk in this demographic. Additionally, although we captured some important data on social vulnerability, we had limited detail about intrinsic structural disparities. For example, age-disparate relationships, intergenerational economic dependence, gender norms, and personal agency to negotiate safer sex practices that could provide deeper insights into the observed disparities in HIV prevalence or the role of education [36, 37]. 

## Conclusions

This study among pregnant women in East London, South Africa, highlights the nuanced relationship between educational attainment, age, and HIV prevalence. Higher educational attainment was strongly associated with lower HIV prevalence, but this positive effect diminished with age. These findings underscore that education alone cannot sufficiently mitigate socioeconomic risks, particularly those shaped by entrenched disparities, historical contexts, and relational power dynamics.

Tailored HIV prevention strategies are essential. For younger women, reinforcing and expanding education-based programs can amplify the favourable effects of schooling, particularly through enhanced sexual and reproductive health agency and awareness. For older women, integrating educational efforts with strategies addressing economic empowerment, healthcare access, and relational dynamics is critical. These interventions should account for historical and contextual influences that may shape risk differently across age cohorts.

Overall, effective HIV prevention in women of reproductive age requires comprehensive, age-targeted approaches that address the evolving vulnerabilities throughout the reproductive lifespan per context. By addressing economic and relational imbalances and adapting strategies to distinct age groups, public health programs may maximize the impact of available resources and control HIV more effectively.
